# Supplementary material for: Optimizing the Composition of the Substrate Enhances the Performance of Peroxidase-like Nanozymes in Colorimetric Assays: A Case Study of Prussian Blue and 3,3′-Diaminobenzidine
Source: Molecules. 2023 Nov 16;28(22):7622. doi: 10.3390/molecules28227622 (PMC10674554; doi:10.3390/molecules28227622)
Supplement: Supplementary file 1 [file molecules-28-07622-s001.zip › molecules-2659131-supplementary.pdf]

Supporting Information

**Optimizing the Composition of the Substrate Enhances the Performance of Peroxidase-like Nanozymes in Colorimetric Assays: A Case Study of Prussian Blue and 3,3'-Diaminobenzidine**

## Materials

Streptavidin was from ProspecBio (Israel). Potassium hexacyanoferrate (III), casein, gelatin A 180 bloom, Proclin-950, hydrogen peroxide were from Sigma-Aldrich (USA). Iron (III) chloride hexahydrate, Tween-20, glutaraldehyde, ammonium chloride, citric acid, TRIS, HEPES, MES, glycine, sodium phosphate, sodium bicarbonate, 3,3',5,5'-tetramethylbenzidine dihydrochloride (TMB), hydrogen peroxide, and glycerol were from ITW (USA). Diaminobenzidine, dialysis tubing (cellulose membrane; 10,000 MWCO) were from Thermo Scientific (USA). Potassium hydroxide, sodium hydroxide, sulphuric acid, and hydrochloric acid were from Reakhim (Russia). Bovine serum albumin was from Biosera (France). Oligonucleotides 5'™-Biotin-GGGGCACGTTTATCCGTCCCTCCTAGTGGCGTGCCCC-FAM-3′ (Bi-D17.4-FAM) and 5'™-$NH_2$-GGGGCACGTTTATCCGTCCCTCCTAGTGGCGTGCCCC-FAM-3′ ($NH_2$-D17.4-FAM) were obtained from Syntol (Russia). Mouse monoclonal IgG2a (against human prostate specific antigen), recombinant spike protein of SARS-CoV-2 were obtained from HyTest (Finland). 96-well polystyrene plates (high binding) were from SPL Life Sciences (South Korea) Nitrocellulose membrane, 0.45 µm pore diameter, was from Bio-Rad (USA). Horseradish peroxidase conjugated with streptavidin (HRP-Str) was from Imtek (Russia). Biotinylation of BSA was performed as described in [27]. Commercial TMB substrate buffer was from anti-Pertussis IgG ELISA kit (Euroimmun, Germany).

Blood serum samples were from our laboratory collection. Serum samples were obtained in 2014 (pre-COVID-19 era) and 2022 (post-COVID-19 era) in the framework of other projects dedicated to the study of post-vaccination immunity. Sera samples were stored at -20 °C. Antibodies against the spike protein of SARS-CoV-2 in these samples were detected by ELISA (Vektor-Best, Russia). Positive (>4000 BAU/mL) and negative sera (no antibodies detected) were pooled and used in dot blot assay. This research was performed according to World Medical Association's Declaration of Helsinki and Council of Europe Protocol to the Convention on

Human Rights and Biomedicine and approved by the Local Ethics Committee. Written informed consent was obtained from all the participants.

Instrumentation. Multiskan Sky UV-Vis Reader was from Thermo Scientific (USA). ZetaSizer NanoZS particle analyzer was from Malvern (UK). VCX-130 ultrasonic processor was from Sonics & Materials (USA). Trans-Blot® SD Semi-Dry Transfer Cell was from Bio-Rad (USA)

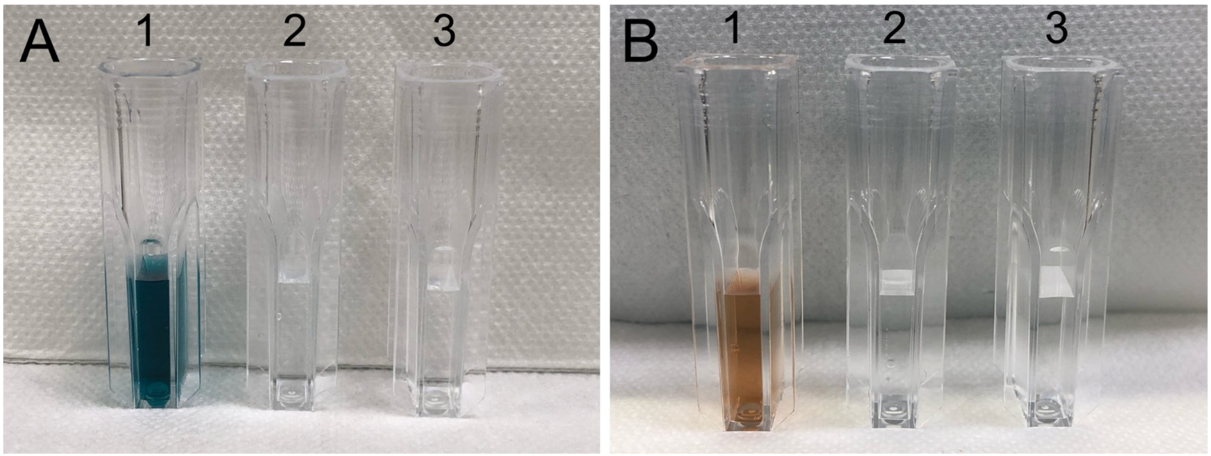


Figure S1. Peroxidase- and oxidase-like activity of nanoparticles. A - TMB substrate, B - DAB substrate. 1 - PB@Gel/Str+$H_2O_2$+TMB/DAB, 2 - PB@Gel/Str+TMB/DAB, 3 - $H_2O_2$+TMB/DAB. Images were taken 10-15 min after mixing of reactants.

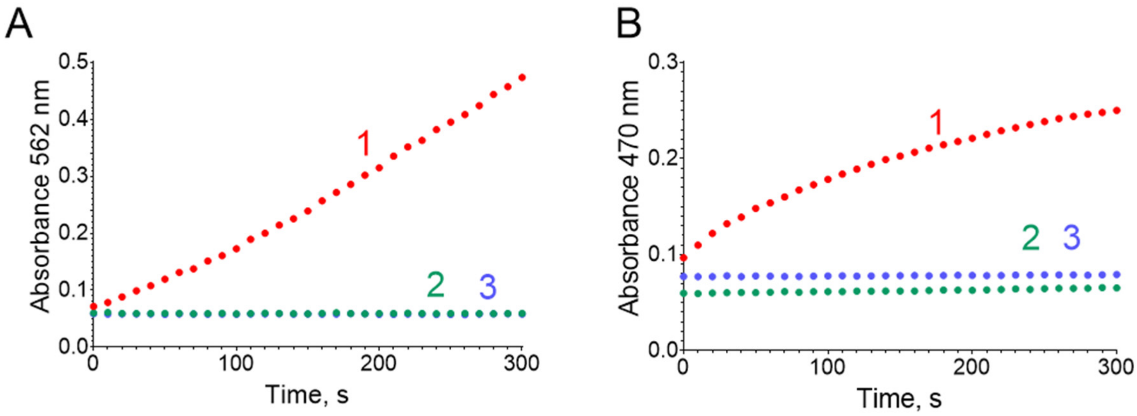


Figure S2. Peroxidase- and oxidase-like activity of PB@Gel/BSA. A - TMB substrate, B - DAB substrate. 1 - PB@Gel/BSA+$H_2O_2$+TMB/DAB, 2 - PB@Gel/BSA+TMB/DAB, 3 - $H_2O_2$+TMB/DAB. Images were taken 10-15 min after mixing of reactants.

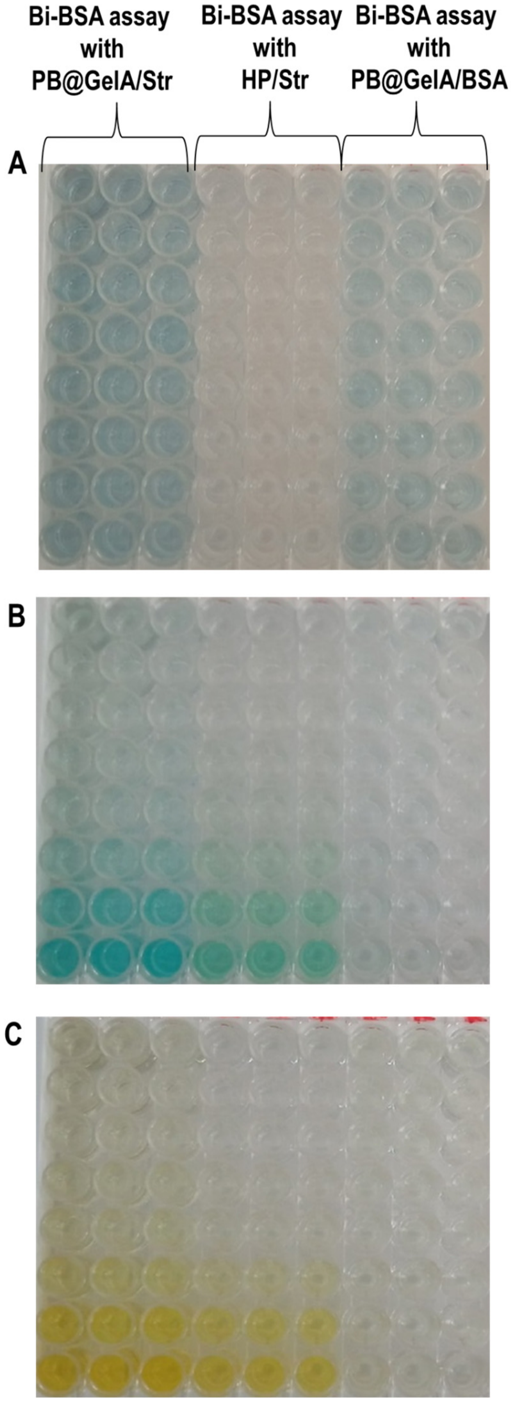


Figure S3. Colorimetric assay for direct detection of Bi-BSA. A) Polystyrene plate filled with conjugates of Prussian Blue nanoparticles and horseradish peroxidase, B) plate after 30 min of reaction with substrate, C) plate after the addition of 2 M $H_2SO_4$. Concentration of Bi-BSA increased from top to bottom

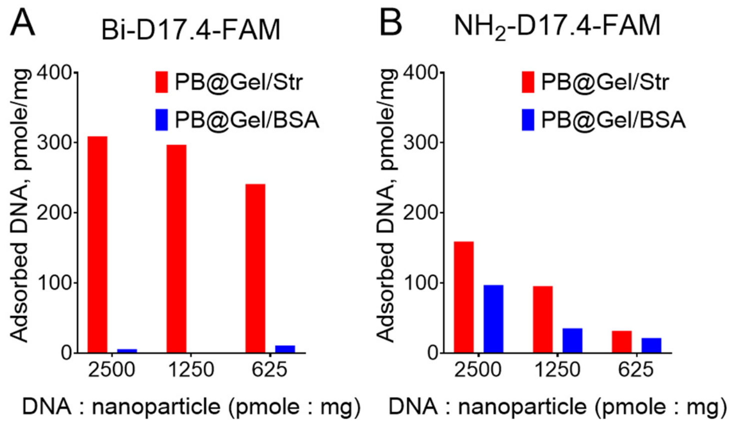


Figure S4. Adsorption of biotinylated and non-biotinylated oligoDNA on PB@Gel/Str and PB@Gel/BSA. Mean values are shown, n=3

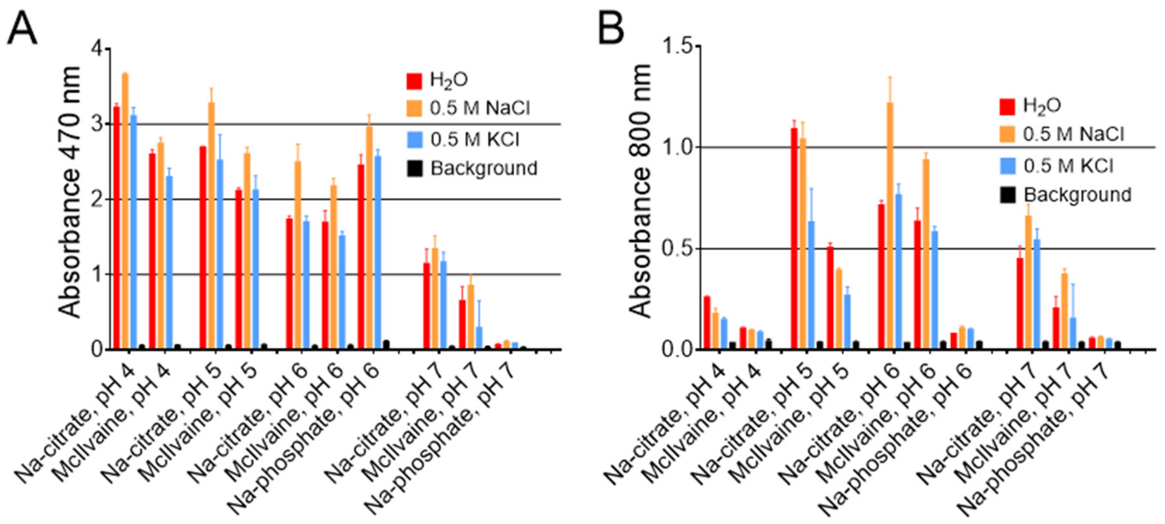


Figure S5. DAB substrate optimization. Absorbance of product at 470 nm (A) and at 800 nm (B). Effect of NaCl and KCl addition. Final concentrations of NaCl and KCl in substrate are given. Molarity of all buffers was 33 mM. Background - substrate solution without salts and nanoparticles. Mean±SD, n=2

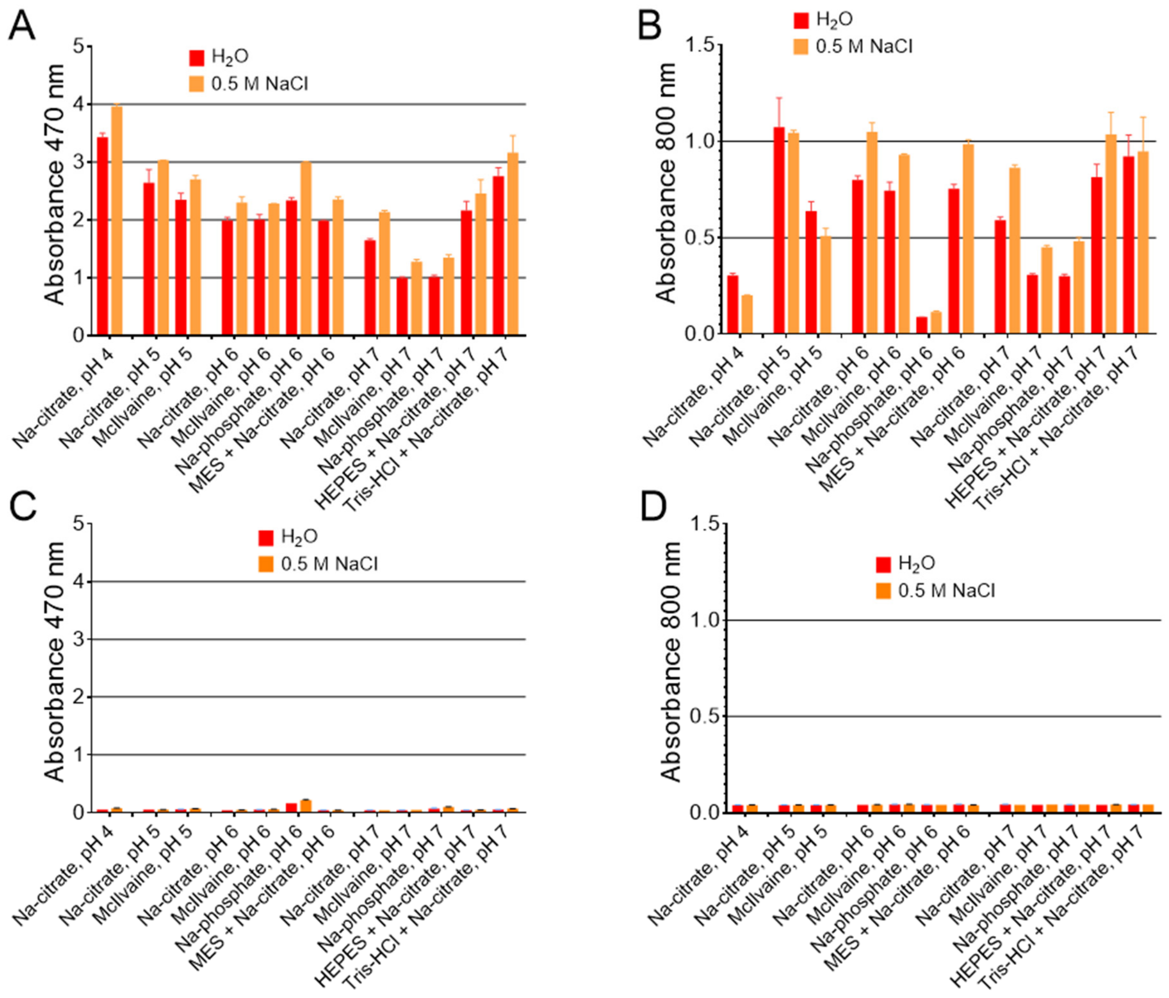


Figure S6. DAB substrate optimization. Effect of NaCl addition. Final concentration of NaCl is given. Molarity of all buffers was 33 mM. 9 parts of MES, HEPES, and TRIS-HCl buffers were mixed with 1 part of Na-citrate buffer with the same pH prior to the experiment. A, B - absorbance after the addition of PB@Gel/Str; C, D - absorbance before the addition of PB@Gel/Str. Mean±SD, n=2

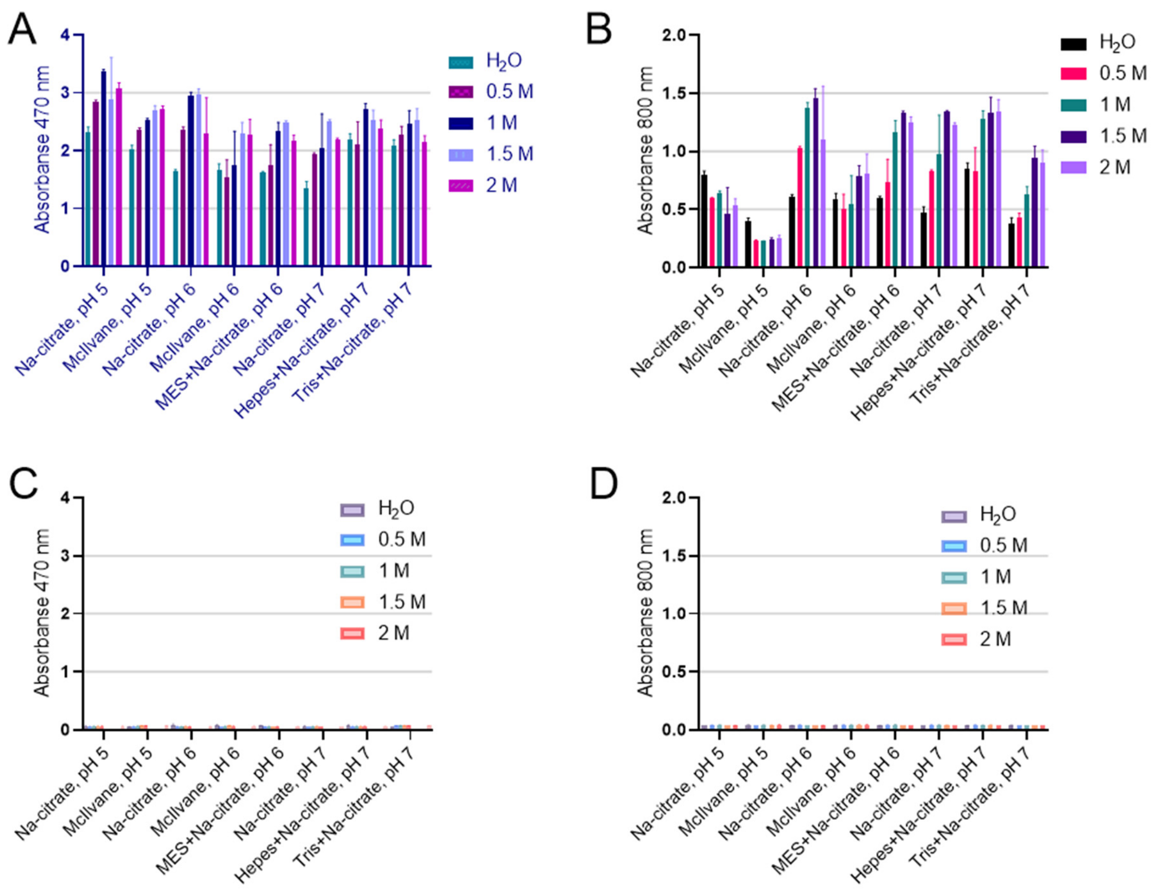


Figure S7. DAB substrate optimization. Effect of $NH_4Cl$ addition. Final concentrations of$NH_4Cl$ are given. Molarity of all buffers was 33 mM. 9 parts of MES, HEPES, and TRIS-HCl buffers were mixed with 1 part of Na-citrate buffer with the same pH prior to the experiment. A, B - absorbance after the addition of PB@Gel/Str; C, D - absorbance before the addition of PB@Gel/Str. Mean±SD, n=2

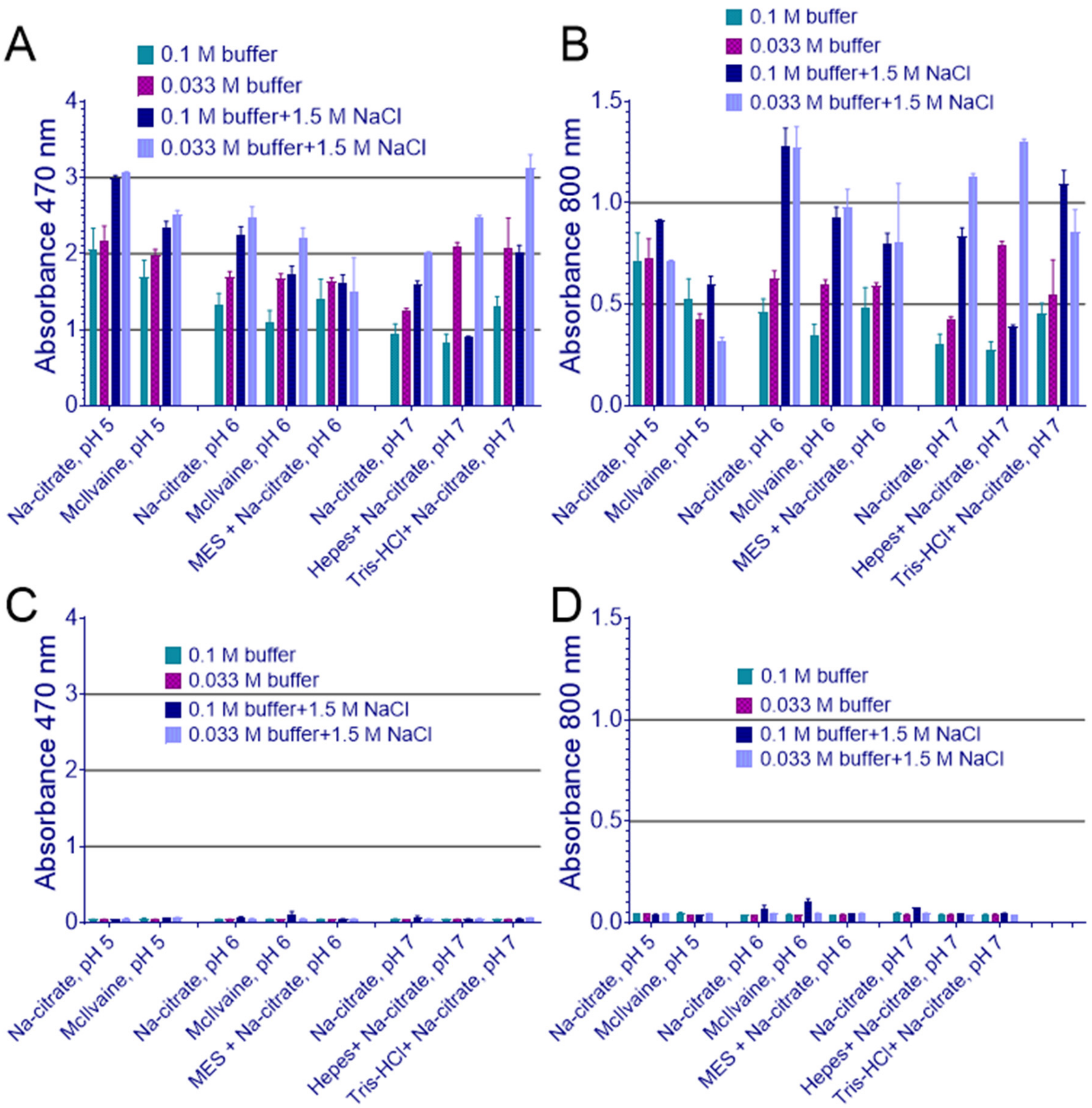


Figure S8. DAB substrate optimization. Influence of buffer molarity and NaCl concentration. Final concentration of NaCl in substrate is given. Final molarities of buffers were 33 or 100 mM. 9 parts of MES, HEPES, and TRIS-HCl buffers were mixed with 1 part of Na-citrate buffer with the same pH prior to the experiment. A, B - absorbance after the addition of PB@Gel/Str; C, D - absorbance before the addition of PB@Gel/Str. Mean±SD, n=2

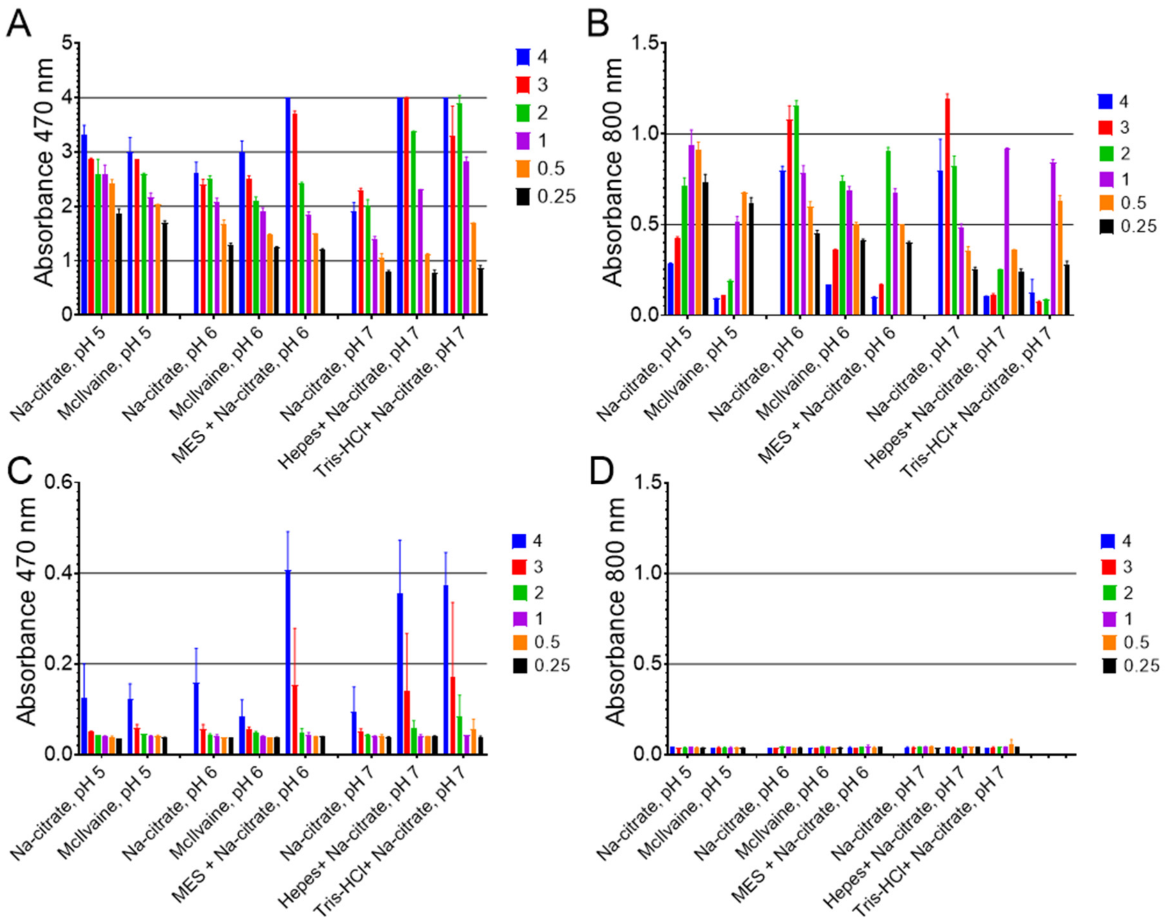


Figure S9. DAB substrate optimization. Influence of DAB concentration. Final concentrations of DAB (in mg/mL) in substrate are given. Molarity of all buffers was 33 mM. 9 parts of MES, HEPES, and TRIS-HCl buffers were mixed with 1 part of Na-citrate buffer with the same pH prior to the experiment. A, B - absorbance after the addition of PB@Gel/Str; C, D - absorbance before the addition of PB@Gel/Str. Mean±SD, n=2

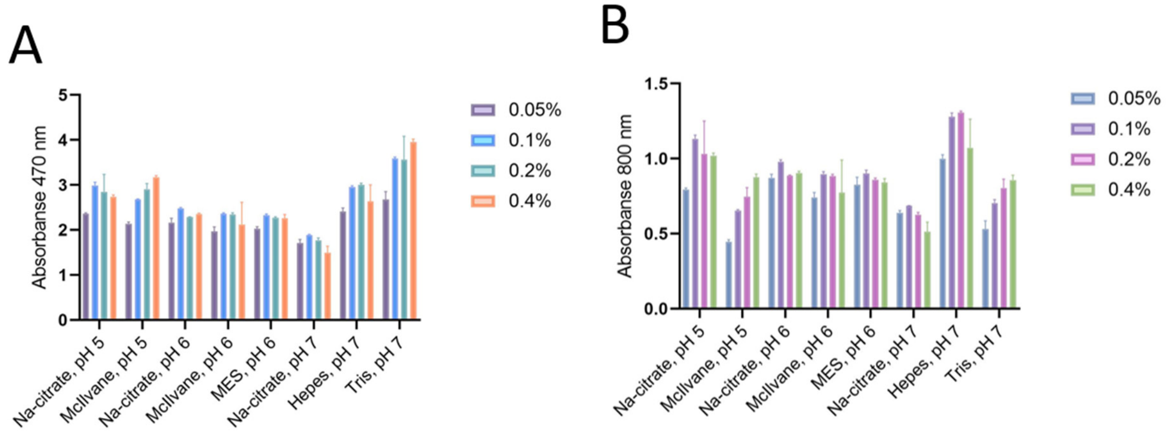


Figure S10. DAB substrate optimization. Absorbance of product at 470 nm (A) and at 800 nm (B). Influence of $H_2O_2$ concentration. Final concentrations of $H_2O_2$ in substrate are given. Mean±SD, n=2

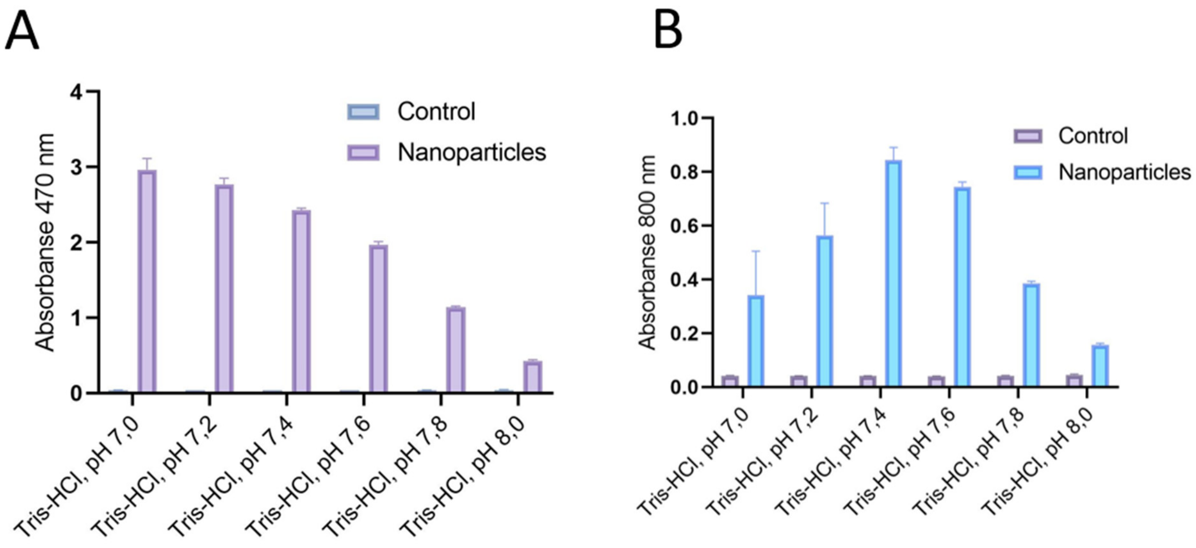


Figure S11. DAB substrate optimization. Absorbance of product at 470 nm (A) and at 800 nm (B). Influence of pH 7.0 - 8.0 Tris-HCl. Molarity of all buffers was 33 mM. 9 parts of Tris-HCl buffer were mixed with 1 part of Na-citrate buffer with the same pH prior to the experiment. Control - absorbance of substrate solution without nanoparticles. Mean±SD, n=2

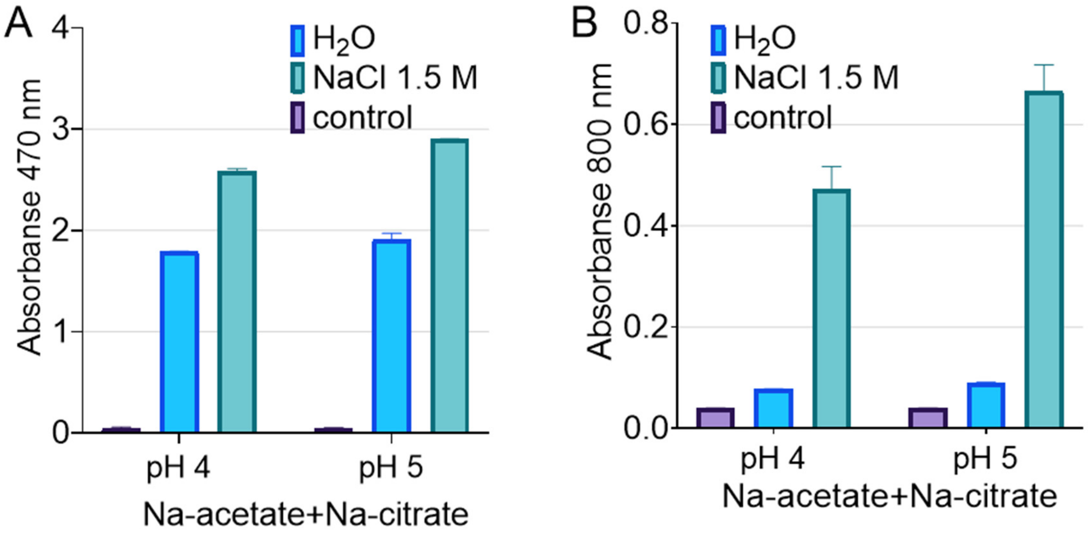


Figure S12. DAB substrate optimization. Absorbance of product at 470 nm (A) and at 800 nm (B). Effect of Na-acetate buffer. 9 parts of Na-acetate buffer were mixed with 1 part of Na-citrate buffer with the same pH prior to the experiment. Control - absorbance of substrate solution with NaCl without nanoparticles. Mean±SD, n=2

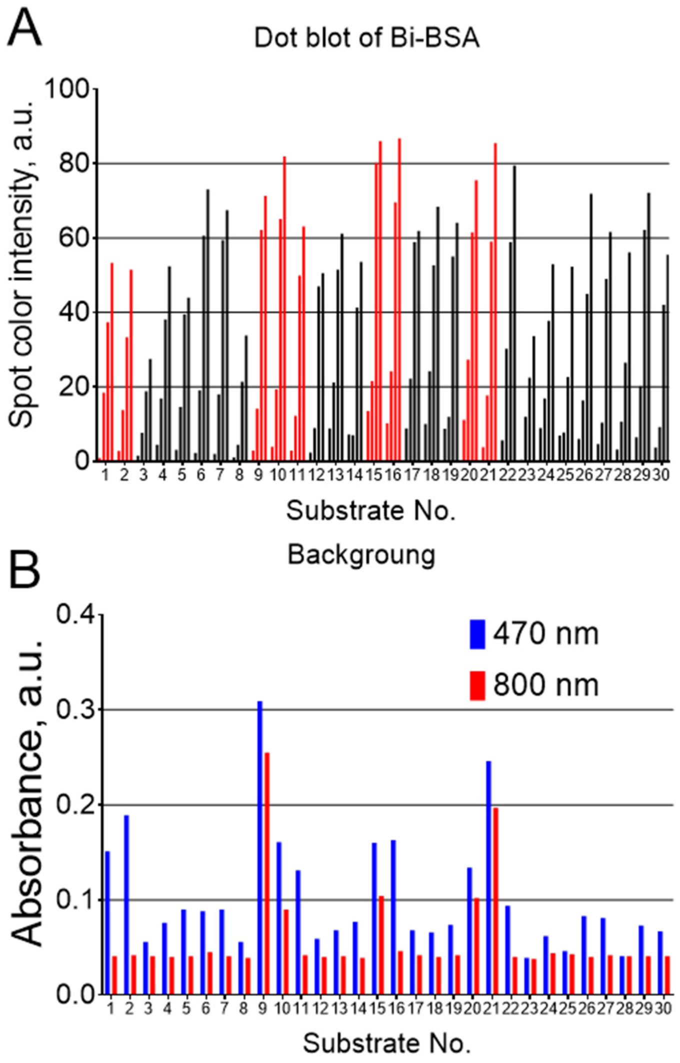


Figure S13. A - Comparison of 30 candidate DAB substrates (see Table S1) using the dot blot assay of Bi-BSA. Four columns for each substrate indicate color intensity for spots with increasing Bi-BSA concentration (from left to right): 0.08, 0.4, 2, and 10 μg/mL. Substrates that produced a high background (>0.1 a.u., see Figure S13B) are marked with red. These substrates were excluded. B - background produced by analyzed substrates. Substrates were prepared and kept for 10 min in 96-well plates without nanoparticles. Absorbance at 470 and 800 nm was then measured. n=1

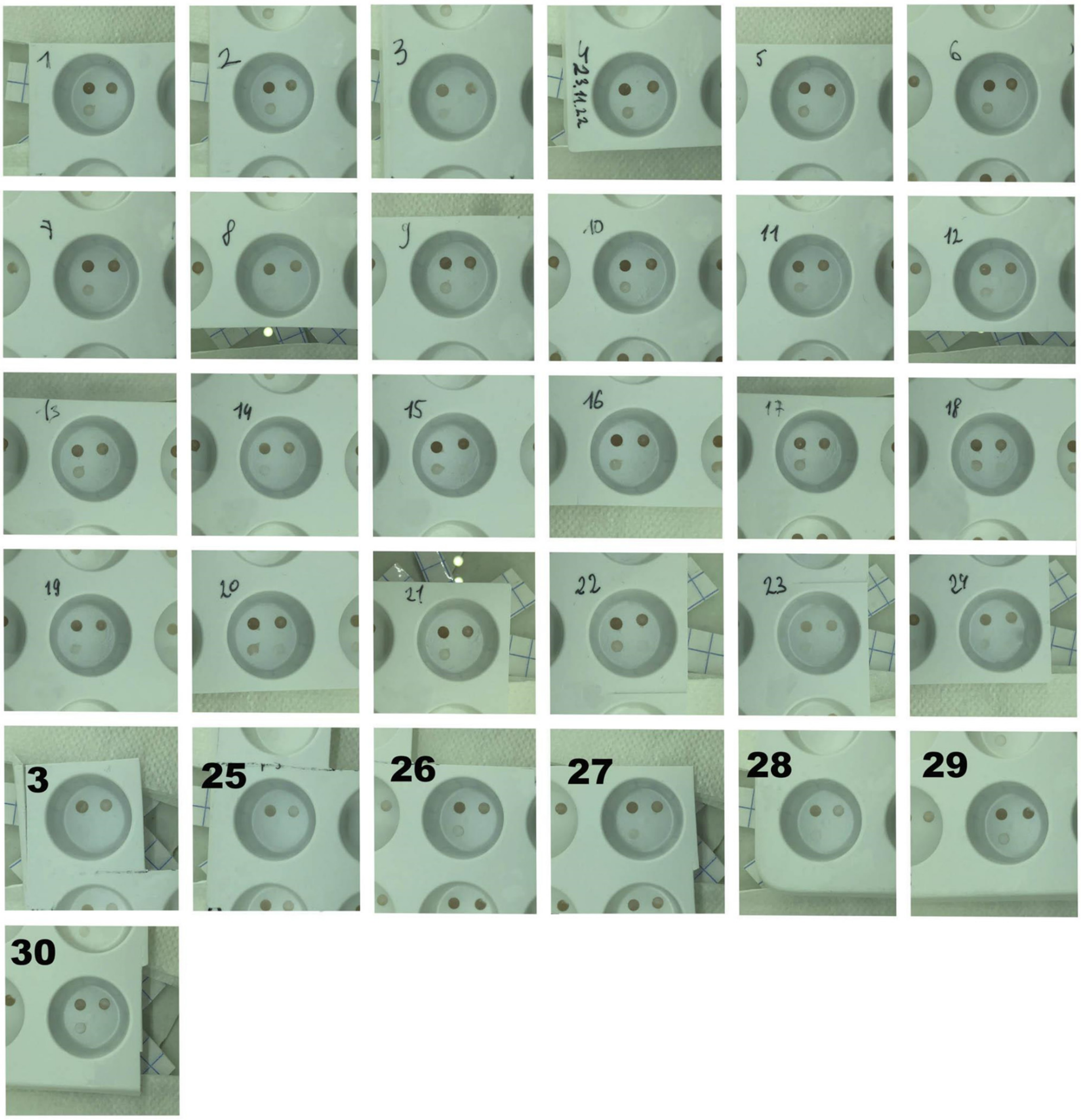


Figure S14. Photos of polystyrene wells after the assay of Bi-BSA with different substrates. These photos were edited: we removed the marks made with a felt-tip pen on the surface of the plates for clarity.

**Image processing**

Segmentation of the images to highlight the area corresponding to the islet was done in Ilastik software. For this purpose, 1-2 images were manually pre-segmented (Figure S15A), the figure below shows the marking process. If the segmentation was not satisfactory (Figure S15B), another image was marked out. As a result, a mask was generated (Figure S15C) containing three types of objects - the islet, the surrounding tissue and the background (the empty part of the image with no tissue).

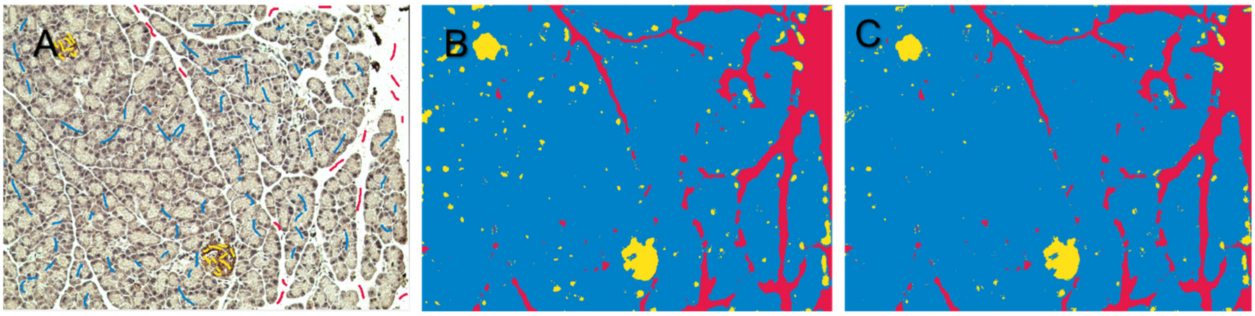


Figure S15. Image segmentation process in Ilastik. A is the original image on which various objects are marked. Yellow is the islet, blue is the surrounding tissue, and red is the part of the image without tissue. B - the image with unsatisfactory segmentation (you can see many objects mistakenly recognized as an island). C - satisfactory segmentation.

The masks obtained, together with the original images, were then processed in CellProfiler. In the first step, the three-component mask (Figure S16A) was divided into individual binary masks corresponding to the islet (Figure S16B), tissue (Figure S16C) and background.

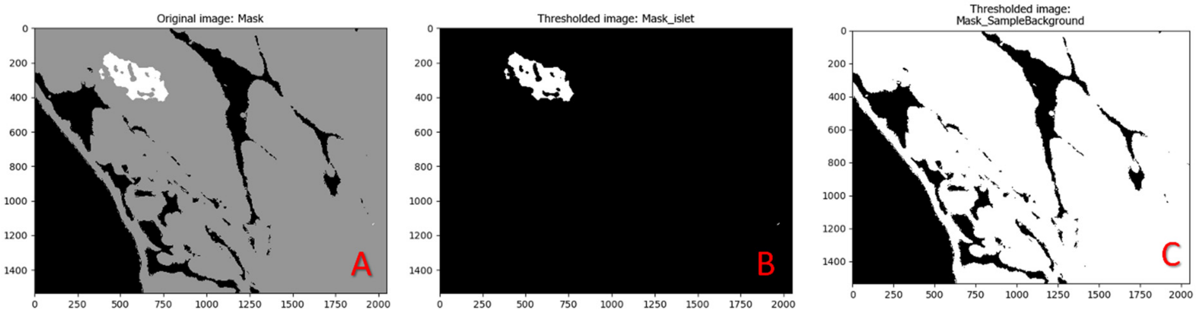


Figure S16. Three-component mask obtained by segmentation(A) and its parts corresponding to the islet(B) and the entire tissue(C).

The mask was then subjected to morphological erosion Figure S17 (to remove small false segmented objects) and dilatation (to remove holes in the mask created by the previous operation).

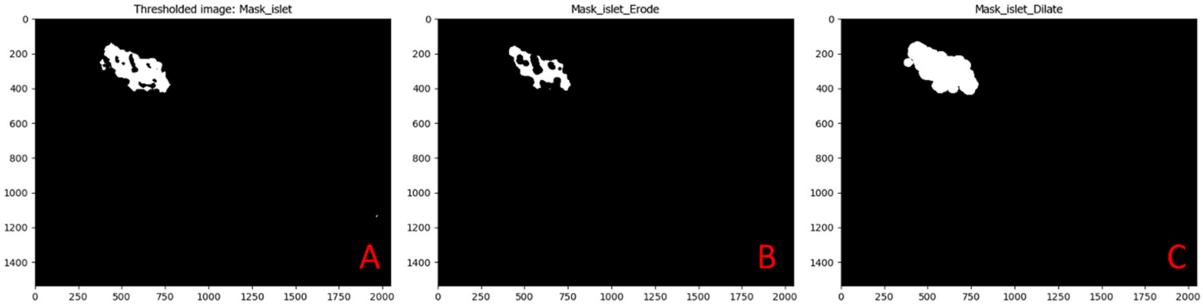


Figure S17. Initial mask (A), mask after morphological erosion(B) and dilatation(C)

The next step was to work with the original image itself. Using the UnmixColors module, the color deconvolution of the image was performed in order to isolate the color channel corresponding to the color of the oxidized DAB(Figure S18).

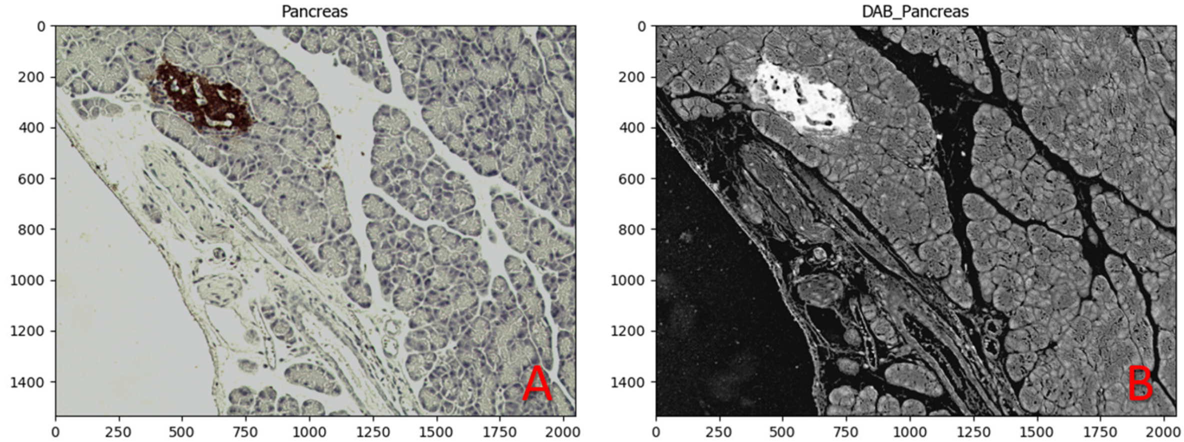


Figure S18. Original image (A) and isolated DAB channel in grayscale (B)

The masks were then applied to the DAB channel image to highlight the islet area (Figure S19B) and the tissue area without the islet (Figure S19C), respectively. Then using the MeasureImageIntensity module we measured color saturation in the corresponding parts of the image.

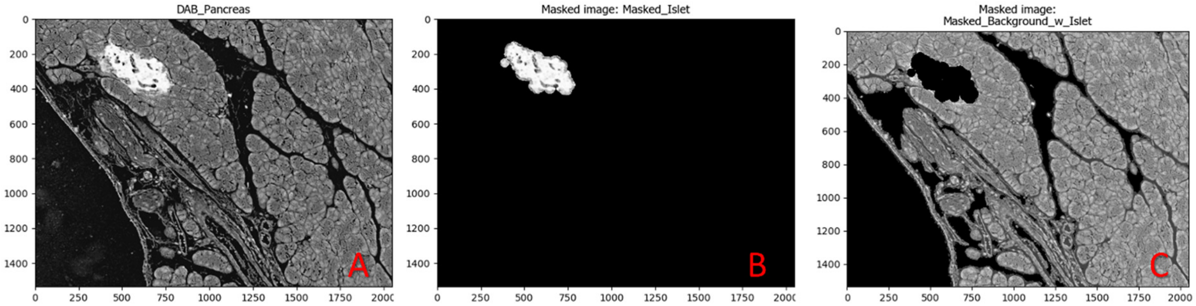


Figure S19. Isolated DAB channel in grayscale (A), islet part of the image (B) and tissue without islet part(C).

The data was saved as a table and further processed in OriginLab.

Table S1. Substrate compositions chosen for dot blot assay. Three best substrates are highlighted.

| No | Buffer | Salt | DAB | $H_2O_2$ |
|---|---|---|---|---|
| 1 | 30 mM Na-acetate, pH 5 + 3 mM Na-citrate, pH 5 | 1.5 M NaCl | 1 mg/mL | 0.1% |
| 2 | 30 mM Na-acetate, pH 5 + 3 mM Na-citrate, pH 5 | 1.5 M $NH_4Cl$ | 1 mg/mL | 0.1% |
| 3 | 33 mM Na-citrate, pH 5 | - | 1 mg/mL | 0.1% |
| 4 | 33 mM Na-citrate, pH 5 | 1.5 M NaCl | 1 mg/mL | 0.1% |
| 5 | 33 mM Na-citrate, pH 5 | 1.5 M $NH_4Cl$ | 1 mg/mL | 0.1% |
| **6** | **33 mM Na-citrate, pH 6** | **1.5 M NaCl** | **1 mg/mL** | **0.1%** |
| 7 | 33 mM Na-citrate, pH 6 | 1.5 M $NH_4Cl$ | 1 mg/mL | 0.1% |
| 8 | 33 mM Na-citrate, pH 6 | - | 2 mg/mL | 0.1% |
| 9 | 33 mM Na-citrate, pH 6 | 1.5 M NaCl | 2 mg/mL | 0.1% |
| 10 | 33 mM Na-citrate, pH 6 | 1.5 M $NH_4Cl$ | 2 mg/mL | 0.1% |
| 11 | 0.3x McIlvaine, pH 6 | 1.5 M NaCl | 1 mg/mL | 0.1% |
| 12 | 30 mM MES, pH 6 + 3 mM Na-citrate, pH 6 | 1.5 M NaCl | 1 mg/mL | 0.1% |
| 13 | 30 mM MES, pH 6 + 3 mM Na-citrate, pH 6 | 1.5 M $NH_4Cl$ | 1 mg/mL | 0.1% |
| 14 | 30 mM MES, pH 6 + 3 mM Na-citrate, pH 6 | - | 2 mg/mL | 0.1% |
| 15 | 30 mM MES, pH 6 + 3 mM Na-citrate, pH 6 | 1.5 M NaCl | 2 mg/mL | 0.1% |
| 16 | 30 mM MES, pH 6 + 3 mM Na-citrate, pH 6 | 1.5 M $NH_4Cl$ | 2 mg/mL | 0.1% |
| 17 | 33 mM Na-citrate, pH 7 | 1.5 M NaCl | 1 mg/mL | 0.1% |
| **18** | **33 mM Na-citrate, pH 7** | **1.5 M $NH_4Cl$** | **1 mg/mL** | **0.1%** |
| 19 | 33 mM Na-citrate, pH 7 | - | 3 mg/mL | 0.1% |
| 20 | 33 mM Na-citrate, pH 7 | 1.5 M NaCl | 3 mg/mL | 0.1% |
| 21 | 33 mM Na-citrate, pH 7 | 1.5 M $NH_4Cl$ | 3 mg/mL | 0.1% |
| **22** | **30 mM HEPES, pH 7 + 3 mM Na-citrate, pH 7** | **1.5 M NaCl** | **1 mg/mL** | **0.1%** |
| 23 | 30 mM Tris-HCl, pH 7.8 + 3 mM Na-citrate, pH 7.8 | - | 1 mg/mL | 0.1% |
| 24 | 30 mM Tris-HCl, pH 7.8 + 3 mM Na-citrate, pH 7.8 | 1.5 M NaCl | 1 mg/mL | 0.1% |
| 25 | 30 mM Tris-HCl, pH 7.0 + 3 mM Na-citrate, pH 7.0 | - | 1 mg/mL | 0.1% |

| | | | | |
|---|---|---|---|---|
| 26 | 30 mM Tris-HCl, pH 7.0 + 3 mM Na-citrate, pH 7.0 | 1.5 M NaCl | 1 mg/mL | 0.1% |
| 27 | 30 mM Tris-HCl, pH 7.0 + 3 mM Na-citrate, pH 7.0 | 1.5 M $NH_4Cl$ | 1 mg/mL | 0.1% |
| 28 | 30 mM Tris-HCl, pH 7.4 + 3 mM Na-citrate, pH 7.4 | - | 1 mg/mL | 0.1% |
| 29 | 30 mM Tris-HCl, pH 7.4 + 3 mM Na-citrate, pH 7.4 | 1.5 M NaCl | 1 mg/mL | 0.1% |
| 30 | 30 mM Tris-HCl, pH 7.4 + 3 mM Na-citrate, pH 7.4 | 1.5 M $NH_4Cl$ | 1 mg/mL | 0.1% |

Table S2. Optimized parameters of DAB substrate

| **Parameter** | **Range** |
|---|---|
| Salt (NaCl, KCl, $Na_2SO_4$, $NH_4Cl$) concentration | 0.5-2 M |
| Buffer molarity | 0.033 and 0.1 M |
| DAB concentration | 0.025-4 mg/mL |
| $H_2O_2$ concentration | 0.05-0.4% |
| pH | 4.0-8.0 |
| Imidazole concentration | 0-10 mM |
| Cations ($Fe^{3+}$, $Mn^{2+}$, $Co^{2+}$, $Cu^{2+}$, $Ni^{2+}$) concentration | 0-0.8 mM |
